# National health research systems in the WHO African Region: current status and the way forward

**DOI:** 10.1186/s12961-015-0054-3

**Published:** 2015-10-30

**Authors:** Joses Muthuri Kirigia, Martin Okechukwu Ota, Marion Motari, Juliet Evelyn Bataringaya, Pascal Mouhouelo

**Affiliations:** Research, Publications and Library Services Programme, Health Systems & Services Cluster, World Health Organization, Regional Office for Africa, Brazzaville, Congo; Health Systems and Services Cluster, WHO Country Office, Kampala, Uganda

**Keywords:** Creating and utilizing resources, Financing, Governance of research for health, National health research systems, Production and use

## Abstract

**Background:**

A number of resolutions of the World Health Assembly and the WHO Regional Committee for Africa call upon African countries and their development partners to make the required investments in national health research systems (NHRS) to generate knowledge and promote its use in tackling priority public health challenges. Implementation of these resolutions is critical for Africa to progress with the rest of the world in achieving the post-2015 health sustainable development goal. This study assesses the current status of some NHRS components in the 47 countries of the WHO African Region, identifies the factors that enable and constrain NHRS, and proposes the way forward.

**Methods:**

To track progress in NHRS components and for comparison, a questionnaire that was used in NHRS surveys in 2003 and 2009 was administered in all 47 countries in the African Region. The national health research focal persons were responsible for completing the questionnaire, which had been hand-delivered to them by the WHO country office staff in charge of research, who also briefed them on the survey, went through the questionnaire for clarity, and sought their informed consent.

**Results:**

All the 47 countries responded to the questionnaire, but some did not answer all questions. Of the countries responding to various questions 49 % (23/47) had a national health research policy; 47 % (22/47) had a health strategic plan; 40 % (19/47) had legislation governing research; 53 % (25/47) had a national health research priority agenda; 51 % (24/47) reported having a functional NHRS and a national health research management forum; 91 % (43/47) had an ethical review committee; 49 % (23/47) had hospitals with ethical review committees to review clinical research proposals; 51 % (24/47) had a scientific review committee; 62 % (29/47) had health institutions with scientific review committees; 83 % (39/47) had a national health research focal point; 51 % (24/47) had a health research programme; 55 % (26/47) had a national health or medical research institute or council; 93 % (41/44) had at least one university faculty of health sciences that conducted health research; and 33 % (15/46) had a knowledge translation platform. Forty-seven percent of countries reported having a budget line for research for health in the ministry of health budget. Between 2003 and 2014, the countries with a functional NHRS increased from 30 % to 51 %.

**Conclusion:**

Compared with 2003 and 2009 surveys, our survey found many countries to have made progress in strengthening some of the functions of their NHRS. However, there remains an urgent need for countries without NHRS to establish them and for others to improve the functionality and efficiency of every NHRS component. This is necessary for the national governments to effectively execute their leadership and governance of NHRS and to create an enabling environment within which research for health can flourish.

**Electronic supplementary material:**

The online version of this article (doi:10.1186/s12961-015-0054-3) contains supplementary material, which is available to authorized users.

## Background

Research for health is crucial for the generation of new knowledge for the improvement of health and health equity of the population. There is a particularly urgent need to optimize the use of health research in the African Region, which accounts for only 13 % of the global population but 31 % of the global burden of disease, and which as a result suffers tremendous socioeconomic development repercussions [1]. In 2012, the African Region lost 660,471,911 disability-adjusted life years (DALYs), of which 65.4 % were from communicable diseases, 25.3 % from non-communicable diseases, and 9.3 % from unintentional or intentional injuries. About 263,164,629 (61 %) of the communicable disease DALYs lost were from HIV/AIDS, lower respiratory infections, malaria, diarrhoeal diseases and preterm birth complications. The 96,185,428 (58 %) lost DALYs associated with non-communicable diseases were attributed to cardiovascular diseases, mental and behavioural disorders, digestive diseases, malignant neoplasms and respiratory diseases [2].

Cost-effective interventions that can lower injury and communicable and non-communicable disease levels exist, but their coverage is very low owing to health systems’ weaknesses [3,4], some of which can be attributed to poor leadership and governance, health workforce inadequacies [1,5], insufficiency of medical products, including vaccines, poor availability and access to technologies and medical devices [1], low financing [6,7], and substandard service delivery [1,4]. The situation is exacerbated by inequities in the distribution of health services within the countries and between population groups. Generally, service coverage is lower among females than males, rural than urban dwellers, lowest than highest wealth quintile, and educated than uneducated women [1].

At present, 91 % of African countries are not on track to achieve the health-related Millennium Development Goals by the end of 2015 [1] and have a low probability of attaining the post-2015 health Sustainable Development Goal unless they can find solutions to the challenges facing their health sector. Research is crucial to understand the magnitude of emerging and re-emerging diseases, disease prevention and management policies, options for improving the cost-effectiveness and coverage of available interventions, and opportunities for developing new tools and products for combatting the old and the new health challenges. In addition, research is essential for identifying solutions to health systems’ weaknesses and monitoring the achievement of health systems’ goals. Having a functioning national health research system (NHRS) is a necessary prerequisite for the generation and utilization of pertinent scientific knowledge in the pursuit of Sustainable Development Goal 3, on ensuring healthy lives and promoting well-being for all at all ages [8].

In 2005, the Fifty-eighth World Health Assembly endorsed the 2004 Mexico Ministerial Summit Statement on Health Research that called upon governments to invest at least 2 % of national health expenditures – and international development agencies to invest at least 5 % of health sector aid – in NHRS strengthening [9]. The 2008 Bamako Call to Action on Research for Health reiterated the commitment to reinforce the systems of research and innovation for health and urged national governments and international development agencies to honour their commitment to the Mexico Summit requirements [10]. The Sixty-third World Health Assembly in 2010 endorsed the WHO strategy on research for health and called upon the Member States to strengthen their NHRS [11].

At the regional level, the WHO Regional Committee for Africa adopted, in 1998, a strategic health research plan for the Region for 1999–2003 [12], and signed the Algiers Declaration on Research for Health in the African Region in 2008 [13], further committing their countries to invest at least 2 % of the national health budget and at least 5 % of external aid for health projects and programmes to strengthening of NHRS.

A few NHRS assessments have been conducted in the region – in 2003, which included 10 countries [14]; in 2009, covering 44 countries [15]; in 2012, in South Africa [16]; and in 2014 in Malawi [17] – but there has been no study to assess the progress all the 47 Member States of the WHO African Region have made in strengthening their NHRS. The study reported herein was conducted in 2014 to generate the latest information on NHRS in all the 47 countries to inform the development of a regional strategy on research for health, which will be presented for adoption to the 65th session of the WHO Regional Committee for Africa in 2015.

The specific objectives of this study were to assess the status of some components of NHRS in all countries of the African Region, identify the factors that enable and constrain NHRS, and generate respondents’ ideas on the way forward.

## Methods

### NHRS conceptual framework

**Figure 1:**
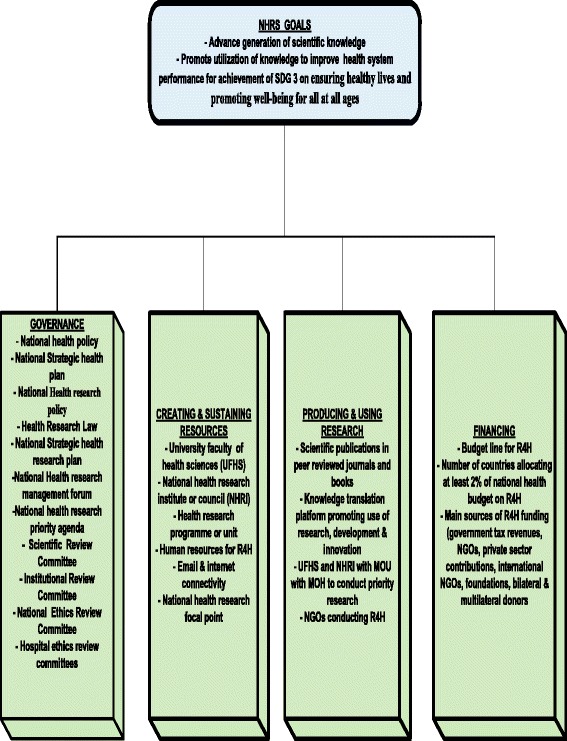
**National health research system conceptual framework.**

A NHRS has been defined as the people, institutions and activities whose primary purpose is to generate scientific knowledge and promote its utilization to improve health and health equity. It consists of the four functions of governance, creating and sustaining resources, producing and using research, and financing. The conceptual framework used for this study, which is presented in Figure 1, has been adapted from Pang et al. [18].

### Governance of research for health (R4H)

The governance of R4H function entails providing oversight of NHRS, including policymaking, creating an enabling research environment, and regulating, monitoring and evaluating R4H performance. For this study, R4H governance includes ensuring the existence and effective functioning of a national health research policy, a national health research plan, health research legislation, a national health research management forum, a scientific review committee, an institutional review committee, and a national ethics review committee. A national health research policy is a formal government statement that defines the R4H vision, priorities and parameters for action in response to health needs, identifying resource and other pressures, and drawn up in close consultation with stakeholders, including communities [19]. A national health research policy document would contain (1) a situation analysis of the burden of disease, socioeconomic environment, health system performance, NHRS performance (including R4H governance, physical infrastructure and human resources, research outputs and extent of their utilization, and finances), and a statement of the major challenges; (2) the long-term vision of the preferred R4H future; (3) guiding principles and values underpinning NHRS; (4) general policy objectives; (5) policy orientations setting out the strategic direction for research, development and innovation; (6) the implementation framework (structures, institutions, strategic partners, communities, civil society and other actors, and their roles and relationships); (7) and monitoring and evaluation mechanisms [20]. Thus, a national health research plan is critically important for R4H direction, coordination of research stakeholders, obviation of fragmentation, and creation of an enabling environment for research and development.

The national health research policy is implemented through the national health research plan. The national health research plan document would contain (1) a background and NHRS achievements; (2) a synthesis of the situation analysis; (3) the strategic NHRS priorities, specifying the strategic directions, specific objectives, targets and main interventions; (4) resource requirements such as human resources, physical infrastructure, equipment, materials and supplies, communication tools, finances and management resources; (5) the finance plan, showing the estimated cost of implementing the strategic directions, available and projected resources, and the financing gap and the ways to close it; (6) the implementation framework, showing the log frame with goals, strategic orientations, objectives, targets, verifiable indicators and means of their verification, the management structure, and stakeholder institutions such as the strategic partners, the civil society, the communities and other actors; and (7) the monitoring and evaluation strategy, showing the proposed mechanism and the costed monitoring and evaluation plan [20].

Health research legislation refers to the statutes, regulations and other legal instruments that are used to protect the human and ethical rights of people participating in research. Legislation is essential for protecting the dignity, safety, health and well-being of research participants. National research for health laws are usually derived from international laws, declarations and guidelines such as the European Charter Patients’ Rights [21], Declaration on Promotion of Patients’ Rights in Europe [22], Revised Lisbon Declaration on Patients’ Rights [23], Convention for the Protection of Human Rights and Dignity of the Human Being with regard to the Application of Biology and Medicine: Convention on Human Rights and Biomedicine [24], World Medical Association Declaration of Helsinki’s Ethical Principles for Medical Research Involving Human Subjects [25], and International Ethical Guidelines for Biomedical Research Involving Human Subjects [26]. R4H law clarifies the responsibilities and accountabilities in the research for all individuals and organizations involved. It is essential for complementing and reinforcing the national health research policy and providing a legal framework that governs the behaviour of all the actors in R4H.

The R4H policy and legal frameworks are implemented through the various R4H governance structures, including the national health research management forum, scientific review committee, institutional review committee, and national ethics review committee. The national health research management forum is an overarching platform with representation of all key research stakeholders, including the heads of animal and human health research institutions, health policymakers, the chair of the parliamentary committee on science and technology, non-profit and for-profit organizations involved in research as users, funders or producers, and representatives of private health service providers, communities, civil society, health-related professional associations, donor community, and the media. The forum ought to be chaired by a government minister such as the minister of health, research or science and technology, and to have the national health research focal person as the secretary. The possible functions of the forum include (1) advising on and developing national health research policies, strategic plans and priorities, and the mechanisms and action plans for their implementation; (2) reviewing the implementation of the national health research strategic plan annually and suggesting strategies to overcome implementation glitches; (3) promoting the development of health research activities internally and externally; (4) recommending the mechanisms to nurture an enabling scientific environment to attract talent and to develop multidisciplinary human resources for research for health; and (5) facilitating utilization of research results [27].

The national scientific review committee is an official organ that reviews each research project’s proposal for scientific rigour and merit, relevance to the national health research priority agenda and adherence to all administrative, safety and legal requirements. It reviews and approves all biomedical or behavioural research proposals involving human research subjects. The committee usually will require the study protocol, lay summary of the protocol, conflict of interest disclosure, informed consent form, supporting documents such as investigators’ curriculum vitae and questionnaires, clinical trial agreement from the sponsor, copy of the grant agreement if the project grant is funded, certification of human subjects for medical eligibility, and research approval form [28]. The institutional review committee plays a similar role but at a specific institution.

The purpose of the national ethical review committee in reviewing research protocols is to contribute to the safeguarding of the dignity, human rights, safety and well-being of all or potential research participants [29]. This committee is vitally important in overseeing adherence to the Council for International Organizations of Medical Sciences’ ethical guidelines for biomedical research to shield human subjects, especially in the Africa, where a significant proportion of the population is vulnerable to exploitation owing to poverty, illiteracy, cultural reverence for ‘healers’ or medical practitioners, high burden of disease and a large unmet healthcare need [30]. These guidelines focus on ethical justification and scientific validity of research, ethical review, informed consent, vulnerability of individuals, groups, communities and populations, women as research subjects, equity regarding burdens and benefits, choice of control in clinical trials, confidentiality, compensation for injury, strengthening of national or local capacity for ethical review, and obligations of sponsors to provide healthcare [26].

### Creating and sustaining resources

This function involves building, strengthening and sustaining the human, physical, institutional and systemic capacities to conduct, disseminate, archive and utilize R4H [18]. In this study, this function is concerned with the health research programme, the national health research institute or council, and universities with faculties of health sciences.

### Production and utilization of health research for health

This function is about producing scientifically valid research outputs, translating and disseminating research, and promoting research use to improve health systems’ performance and tackle the social determinants of health. In regard to this function, this study is concerned with the number of publications in peer-reviewed journals, and the existence of a knowledge translation platform, of non-governmental organisations (NGOs) conducting R4H, and of memorandums of understanding involving the ministry of health, the national health research institute, and universities with faculties of health sciences.

### Financing for R4H

This function concerns the raising of funds for R4H, their pooling and their efficient, transparent and accountable allocation to individuals, institutions and organizations whose primary objective is to produce, disseminate, archive and promote the use of R4H. Part of the mandate under this function involves tracking the flow of R4H resources from the public, private, NGO and philanthropic organizations through the financing intermediaries to the institutions undertaking R4H, and then to the R4H programmes and inputs, including research and development (R&D) expenditures such as those associated with personnel and facilities. R&D expenditures include all current costs and capital expenditure for R&D performed within and outside a statistical unit or sector of the economy in a year. R&D personnel levels are measured in terms of number of person-years used for R&D in a year. R&D facilities include the standardized equipment, library facilities, laboratory space, journal subscriptions, and standardized computer time used per year for R&D work [31,32]. Herein, the components considered under the financing function are the budget line for R4H, the number of countries allocating at least 2 % of their health budget to R4H, and the main sources of R4H funding.

### Data

A survey was conducted between March and November 2014 in all the 47 Member States of the African Region to assess the status of NHRS. For temporal comparison, we used the questionnaires used by Kirigia and Wambebe in 2003 [14] and Mbondji et al. in 2009 [15] to collect the data for the study reported here.

A preamble was added to the old questionnaire that stated that the purpose of the questionnaire was to gather information on the status of NHRS in each country for use in the revision of the expired strategic health research plan of the WHO African Region [12]. Definitions of NHRS and its functions were provided as well as of the various terminology, programmes and institutions mentioned in the questionnaire.

The questionnaire had questions grouped in 10 categories: health research policy, health research legislation, strategic health research plan, research coordination mechanisms, health research programme, research institutes, national universities, health research financing and budget, NGOs involved in health research, and actions needed to strengthen health research capacity. The questions can be grouped under the four functions of NHRS.

### Governance of research for health

The health research policy questions sought to establish whether the country had an official national health policy and strategic health plan and a valid official health research policy. The health research legislation component contained questions on the existence of legislation for health research, whether it included ethical concerns, and the year of its promulgation. The strategic health research plan component had questions on whether a valid strategic health research plan existed, its title and whether it was being implemented.

The research coordination mechanisms component asked questions on whether the country had a functional NHRS, a national health research management forum, a national ethical review committee, a scientific review committee, institutions with institutional review committees, hospitals with ethical review committees to handle clinical research proposals, a national health research focal point, and guidelines for the development of collaboration agreements on health research involving foreign institutions and agencies.

### Creating and retaining resources

The health research programme component consisted of questions on whether the programme had a clearly defined mission statement, terms of reference and organizational structure, and a plan of action, if each technical staff had a computer, if it had e-mail and Internet connectivity, and if it undertook any research by itself. Also sought were the name of the government ministry or department where it was housed, the number of technical and support staff in it, and the titles of the studies it had undertaken in the previous year.

The research institutions section had questions on the existence of a national health research institute or council, the contact details of its director and the number of its personnel. Also sought was whether there was a memorandum of understanding (MoU) between the ministry of health (MoH) and the research institute, the ways in which the institute disseminated its research, and the five key enabling and constraining factors for health research in medical research councils or health research institutes.

The national universities questions sought details on national universities with faculties of health sciences, whether these faculties conducted research, and if they had MoUs with MoH and if the MoUs were for the development of human resources, provision of technical support to MoH, or undertaking of research for MoH. Also sought were the five key enabling and constraining factors for health research at universities.

### Production and utilization of health research for health

Questions on this function asked whether the country had a platform for translating, synthesizing and communicating research to inform health policy and practice, if the health research programme undertook research by itself, if there was an MoU between the MoH and the institute, if the university faculties of health sciences each had an MoU with the MoH for undertaking research for the MoH, etc. Information was sought on the ways in which the heath research institute disseminated its research and the faculties of health sciences conducted research, and on the existence of NGOs in the country that undertook health research.

### Financing for health research for health

The health research financing and budget section had questions on the existence of a budget line for health research in the MoH budget document, the amount of money allocated by MoH to health research in the last financial year (2012/2013), the MoH overall budget for the previous financial year (2012/2013), and the ranking of the different sources of financing for health research in the countries, i.e. government tax revenues, private sector companies, multilateral and bilateral donors, local NGOs, international NGOs, and others. For NGOs involved in health research, respondents were asked whether the country had NGOs that undertook health research, and to include their names, contact information and sources of funding for the research.

The last section of the questionnaire asked the respondents to indicate the actions that could be taken at local and international levels to stimulate health research.

The questionnaire was available in English, French and Portuguese, the three WHO working languages in the African Region. Among the 47 Member States, French is the official language for 21, English for 21, and Portuguese 5. The questionnaire was sent by e-mail to the 47 WHO country offices for onward delivery to the national health research focal person in each of the countries, irrespective of the government ministry where they were housed, who had the primary responsibility for completing the questionnaire. The WHO country office national professional officers in charge of research briefed the national focal points on the purpose of the survey, went through the questionnaire with them, and informed them of their ethical right to participate or not in the survey. The information required was not centralized at the MoH, so the research focal person obtained it from the various sources such as the national health research institutes, national universities’ faculties of health sciences, NGOs that undertook health research, etc.

The data were entered into an Excel spreadsheet and analysed with STATA statistical software.

### Ethical clearance

Ethical clearance for this study was granted by the WHO African Regional Office’s Ethics Review Committee in 2014.

## Results and discussion

### Response rate

All 47 Member States in the Region responded to the questionnaire, but some of them did not answer all the questions and therefore the denominator for some questions is not 47. The high response rate is attributed to the fact that those responsible for completing the questionnaire were in charge of health research in their countries and understood the importance of the survey: they were aware that the information would be vital in the development of a regional WHO strategy on research for health. The close follow-up by the WHO country offices was also a factor in the good response.

### Governance of research for health

Additional file 1 shows the status of R4H governance structures in the 47 countries in the African Region, specifically the national health policy, the national strategic plan for health, the national health research policy, the national strategic health research plan, and relevant health research law.

### Policy and strategic plan

Overall, 45 (96 %) countries reported that they had an official national health policy and 44 (94 %) a national strategic plan for health, whereas 23 (49 %) had an official national health research policy, 22 (47 %) had a national strategic health research plan, 8 of which were implementing it, and 25 (53 %) had a national health research priority list (agenda). An implication of this is that it might be extremely difficult for the 22 countries without a national health research agenda to effectively perform their governance role of ensuring that all health research actors harmonize and align their research with the national health priority needs.

### Legislation

In total, 19 (40 %) countries had a law relating to health research, all of which included ethical concerns. Given the growing volume of clinical trials and medical technology transfer to African countries, it is of great concern that 28 (60 %) countries do not have a law regulating research, development and innovation. For such countries, the dignity, safety, health and well-being of research participants are at risk [30].

### Research coordination and ethical and scientific regulation mechanisms

The existence of both a functional NHRS and a national health research management forum was reported by 24 (51 %) countries; equally, 24 (51 %) countries had a functional scientific review committee and 29 (62 %) an institutional review committee. On the other hand, 43 (91 %) countries had a functional national ethics review committee and 23 (49 %) had hospitals with an ethical review committee for clearing all clinical research proposals. Finally, 39 (83 %) countries had a national health research focal point and 15 (32 %) reported having guidelines for collaboration agreements on health research involving foreign institutions and agencies.

Comparing the results of the current and the past two NHRS surveys [14,15], it appears that R4H governance has improved (Table 1). For example, between 2009 and March 2014, the WHO African Region experienced a growth of 5 % in countries with valid official national health policies, 6 % in countries with a valid strategic health plan, 19 % in countries with an official health research policy, 24 % in countries with a law regulating R4H, 27 % in countries with a national strategic health research plan, 11 % in countries reporting a functional national health research system, 7 % in countries reporting to have a national health research focal point, and 16 % in countries with a national ethics review committee.

**Table 1:** **Trend of NHRS development in the WHO African Region**

**Variable**	**Percentage of countries in the 2014 survey (n = 47)** ^**a**^	**Percentage of countries in the 2009 survey** [15] **(n = 44)** ^**b**^	**Percentage of countries in the 2003 survey** [14] **(n = 10)** ^**c**^
**Governance of NHRS**			
Valid official national health policy	96 % (45/47)	90.7 % (39/43)	70 % (7/10)
Valid strategic health plan	94 % (44/47)	88.1 % (37/42)	80 % (8/10)
Official health research policy	49 % (23/47)	30.2 % (13/43)	30 % (3/10)
Law regulating R4H	40 % (19/47)	16 % (7/44)	10 % (1/10)
National strategic health research plan	47 % (22/47)	20.5 % (8/39)	20 % (2/10)
Functional national health research system	51 % (24/47)	40 % (16/40)	30 % (3/10)
National health research focal point	83 % (39/47)	76.2 % (32/42)	80 % (8/10)
National ethical review committee	91 % (43/47)	75 % (33/44)	60 % (6/10)
**Creating and sustaining R4H resources**			
University/colleges of health sciences conducting research	93 % (41/44)		30 % (3/10)
National health research institute or council	55 % (26/47)		30 % (3/10)
Health research programme at MoH	51 % (24/47)	25 % (11/44)	20 % (2/10)
Average number of researchers in a R4H programme	20.9		7.5
Non-governmental organizations conducting R4H	65 % (30/46)		70 % (7/10)
**Producing and utilizing R4H**			
R4H programme action plan	67 % (16/24)	(8/11)	20 % (2/10)
Existence of a knowledge translation platform	33 % (15/46)		30 % (3/10)
Existence of a health research management forum	51 % (24/47)	24.3 % (9/37)	20 % (2/10)
**Financing R4H**			
Existence of a budget line in the health budget for research for health	47 % (22/47)	41 % (18/44)	40 % (4/10)
Progress towards the target of allocating 2 % of national health budget to R4H	17.6 % (3/17)	4.5 % (2/44)	0 % (0/10)

NHRS, National health research systems; MoH, Ministry of Health; R4H, Research for Health.

^a^Current survey.

^b^Mbondji et al. [15].

^c^Kirigia and Wambebe [14].

### Creating and sustaining resources

Overall, 24 (51 %) countries had a health research programme, 88 % of which were housed under the MoH, 8 % under the ministry of science and technology, and 4 % under the national health research organization. Of the countries with a health research programme, 67 % had a clearly defined mission statement, 79 % had clear terms of reference, and 83 % had an organizational structure. The programmes had a median of 5 (and average of 21) technical and support staff, and 71 % of the technical staff had a computer. All health research programmes had e-mail and Internet connectivity, meaning that staff had access to the latest R4H articles and books and could easily submit manuscripts to online journals for publication.

The presence of a national health research institute or council was reported by 26 (55 %) countries; 44 (98 %) of 45 countries that responded on this question reported having at least one university faculty of health sciences. The average number of university faculties of health sciences per country was 3 (the median was 2), with a standard deviation of 2.7. Sao Tome and Principe did not have a faculty of health sciences.

Between 2003 and March 2014, the WHO African Region experienced a 63 % growth in the number of countries with at least one university college of health sciences conducting research, 25 % in the number of countries with a national health research institute or council, 31 % in the number of ministries of health with a R4H programme, and 179 % in the average number of researchers in a R4H programme [14,15].

### Producing and utilizing research

In terms of production of knowledge, 38 % (9/24) of the health research programmes undertook research themselves, whereas 93 % (41/44) of the countries with at least one university faculty of health sciences reported they were conducting research for health and 65 % (30/46) of the countries had at least one NGO undertaking health research.

Regarding knowledge utilization and management, 33 % (15/46) of the countries had a platform for translating, synthesizing, and communicating research to inform health policy and practice; 54 % (14/26) of the national health research institutes or councils had a MoU with the MoH and 34 % (15/44) of the university faculties of health sciences had a MoU with the MoH for undertaking research for the MoH, among others.

Table 2 shows the ways through which the national health research institutions disseminate research. The five main methods are peer-reviewed articles in journals (69 %), national health research forums and international conferences (58 %), published annual progress reports of the institutions (31 %), web sites, including the Virtual Health Library (19 %), and print and electronic mass media (19 %).

**Table 2:** **Ways that national health research institutes disseminate research**

**Dissemination mechanisms**	**Count**	**Precent**
Peer-reviewed publications in journals	18	69.2
National health research forum and international conferences and seminars	15	57.7
Published annual progress reports of the institutions and/or Ministry of Health report	8	30.8
Feedback meeting with study subjects (community) and district health management teams	1	3.8
Institutional newsletters/posters/brochures in official and local languages	2	7.7
Exhibitions by the division of health research	3	11.5
Website (including virtual health library)	5	19.2
Print and electronic mass media	5	19.2
Research open day	3	11.5
Validation workshop and seminars	2	7.7
In-house scientific committee of researchers at the home institute	1	3.8

Between 2003 and March 2014, the Region registered a 47 % increase in the number of R4H programmes with an action plan, 3 % in countries with a knowledge translation platform, and 31 % in countries reporting having a health research management forum [14,15]. Uthman et al.’s PubMed search [33] revealed that the total number of articles published in the African Region increased from 3,623 in 2000 to 12,709 in 2014, indicating a relative growth of 251 %. The percentage share in the worldwide research publications per year increased from 0.7 % in 2000 to 1.3 % in 2014. In spite of the growth in the total published R4H articles, the African Region accounts for a very low output in the global R4H publishing.

### Financing of research for health

In total, 47 % (22/47) of the countries reported having a budget line for health research in the MoH budget document. The most important method of financing R4H was international NGOs followed by government tax revenues (Table 3).

**Table 3:** **Most important sources of funding for health research**

**Source of funding**	**Number of countries**	**Percent**
International NGOs	16	45.7
Multilateral and bilateral donor funding	3	8.6
Government tax revenues	6	17.1
Private sector companies	2	5.7
Local NGOs	4	11.4
Other	4	11.4
Total	35	100

During the period 2009 to March 2014, the Region registered a 6 % increase in the number of countries with a budget line for R4H in the health budget, and 13 % in the number of countries allocating at least 2 % of their health budget to R4H.

### The way forward through the respondents’ eyes

The respondents made several suggestions for action at local and international levels to strengthen NHRS.

### Governance of R4H

Five actions were recommended for national governments to strengthen R4H governance. First, the overall R4H design and oversight should be bolstered through strengthening or building institutional capacity to govern, coordinate and oversee health research, including instituting better management of human, material and financial R4H resources (Table 4). To stimulate and ensure R4H coordination, it was suggested that a functional national R4H forum or committee be created, R4H activities be decentralized to lower administrative levels, including designating or hiring R4H focal persons for those levels, and that the development of standard operating procedures and other guiding reference documents for use by the scientific, institutional, national, and hospital ethics review committees be supported.

**Table 4:** **Actions for local and international levels to reinforce governance of R4H**

**Interventions by African governments**	**Number of countries**
Establish NHRS structures and build institutional capacity to govern, coordinate and oversee health research, including for better management of human, material and financial resources	30
Develop/launch a national policy on health research	14
Ensure observance of ethics and good practices, including regulation of research through the national ethics review committee and institutional ethics review committees	9
Establish a national health research forum or committee to support health research	8
Develop specific national legislation for health research	8
Promote multisectoral and multidisciplinary health research and promote community participation in health development research	6
Build networks of researchers and research centres and strengthen partnerships between research institutes and universities with faculties of health sciences	3
Define a national priority research agenda for the coming 3 years to ensure that research undertaken is harmonized and addresses tangible prioritized country needs and challenges	3
Support the development of standard operating procedures and guidance reference documents	2
Decentralize research for health activities to the counties, including hiring research focal persons at the decentralized or devolved levels	1
Establish a system for monitoring and evaluating research	1
**International community interventions**	**Number of countries**
Facilitate networking of institutions for health research (for example, for undertaking multi-country studies), international cooperation and partnership; advocate for formal partnerships between universities and research institutions	12
Provide technical support for the development and implementation of national health research policy and strategic plan, and ensure externally driven research is aligned with national research priorities	10
Provide technical support for the development of legislation on health research	7
Support the development of the national health research agenda or priority list and their alignment, and cooperate with researchers and other partners to implement and evaluate them	6
Support country and intercountry research collaboration and coordination	4
Provide technical support for establishment of a national committee of ethics and training of its members	2
Support capacity strengthening for laboratories for their accreditation through providing equipment, infrastructure and logistics support	2
Develop internationally recognized regulatory standards, policies and guidelines and foster comparable research in health	2
Support evaluation of research and development	1

Second, a policy framework should be developed and implemented, covering the national R4H policy, strategic plan and priority research agenda, and spanning several years to ensure that the research undertaken is harmonized and addresses tangible, prioritized, country-specific public health needs or challenges. Third, the safety and dignity of research subjects and researchers should be assured through developing and enforcing an R4H law to regulate research, defining and applying ethical standards for R4H and research partnerships, and creating functional research ethics review committees at the national and institutional levels to ensure good ethical practices. Fourth, a multisectoral and multidisciplinary research environment should be created and promoted through building and nurturing networks of researchers and research centres, strengthening partnerships between research institutions and universities with faculties of health sciences, and promoting community participation in R4H. Fifth, a system for monitoring and evaluating the performance of NHRS should be established.

A number of actions were recommended for the international community to buttress African countries’ R4H governance role: (1) to provide technical and financial support for the development and implementation of the national R4H policy, strategic plan and priority agenda; (2) to support the institutionalization and alignment of the national health research agenda, cooperating with researchers and other partners to implement and evaluate it; (3) to support country and intercountry research collaboration and coordination; (4) to advocate for and foster formal partnership between local universities and research institutes, south-south and north-south partnership, and networking of institutions on R4H, including encouraging the conduct of multicountry studies; and (5) to provide technical and financial support for the development and implementation of a regulatory framework to guide the behaviour of individuals and organizations charged with the creation and management of human, material, infrastructural and systemic resources for R4H, production of research, and mobilization, disbursement and accountability for R4H financial resources.

The actions suggested for international actors for augmenting the R4H regulatory capacity include support for the development of R4H legislation, establishment of national ethics review committees and strengthening of the capacity of the members, strengthening the capacity of research laboratories for their accreditation, and defining and enforcing international research and ethical standards. Further, it is necessary to provide support for evaluation of research and development.

### Creating and sustaining resources

Table 5 shows the actions recommended by the respondents for local and international levels to strengthen the creation and sustenance of R4H human resources and institutions that produce R4H resource inputs and those that combine those inputs to conduct, absorb and utilize research. These inputs include physical facilities such as offices, laboratories, equipment, devices, computers and peripherals, vehicles, and information and communication technologies needed to conduct, absorb, and use research findings.

**Table 5:** **Actions at local and international levels to reinforce creation and sustenance of R4H resources**

**Interventions by African governments**	**Number of countries**
Strengthen master’s and PhD level research human resource levels through retention and motivation approaches and enhancing competencies and other capacities. For example, provide bursaries especially at postgraduate level or for young researchers, draw up career development paths for research staff (including financial management) and give priority to researchers and research teachers	34
Establish a national health research institute or council	7
Build or strengthen research infrastructure, such as offices and laboratories, and provide equipment, reagents and programme vehicles	6
Establish/activate a national health research programme, unit or department	9
Ensure official confirmation of the national focal point for health research	2
**Actions at the international level**	**Number of countries**
Support strengthening of human resources for health research capacity through training and retention initiatives such as exchange programmes and scholarships for postgraduate and specialized training	18
Provide technical support for the establishment of the R4H programme	2
Create international research observatories for the compilation and analysis of data at the international scale	1
Provide technical support for the establishment of a national health research institute	1

A number of actions were recommended for national governments to strengthen the creation and sustenance of R4H resources. First, the production and retention of a critical mass of human resources for health research should be facilitated. The governments should increase the numbers of master’s and PhD level staff, provide staff retention-motivation incentives, and strengthen staff competencies and other capacity through training by offering bursaries or training grants especially at the postgraduate level and for young researchers. In addition, remuneration for research for health staff should be prioritized and their career development paths clearly elaborated. Second, an infrastructural enabling environment should be created, which partly entails establishing or reinforcing a national R4H institute or council and a national R4H programme, unit or department. Third, a national focal point for R4H should be designated officially. This will be the contact point for all matters related to health research. The need to provide decent offices equipped with computers, printers and information and communication technology connectivity, programme vehicles, and adequate laboratory infrastructure, equipment and reagents was underscored.

Five actions were proposed for the international community to complement those of the national governments in the creation and sustaining of the resources for R4H. First, they should support the production of master’s degree and PhD level researchers, retention and motivation of staff, and updating and retention of competencies and other capacities through awarding of bursaries especially at the postgraduate level. Second, they should support the strengthening of health research human resources through training and other capacity strengthening approaches, including student and faculty exchange programmes and scholarships for postgraduate and specialized research training. Third, they should provide technical, financial and material support for the establishment and bolstering of national health research institutions. Fourth, they should support the establishment and reinforcement of R4H programmes. Fifth, they should create international research and development observatories for the compilation and analysis of R4H data on an international scale.

### Producing and using research

Research outputs and their use in improving population health are the fruits of a vibrant NHRS. Table 6 presents the actions recommended by the respondents for the national and international levels to boost the production and utilization of R4H. Five recommendations were made for the national level. First, there is need to develop a research communication and translation platform to facilitate the use of research findings for policy (and decision-making), practice and product development. National governments can leverage the learning from the WHO initiative called Evidence Informed Policy Networks (EVIPNet) to develop their contextualized knowledge translation platform to facilitate translation of research findings into actions.

**Table 6:** **Actions recommended by respondents for national and international levels to increase production and use of R4H**

**Country actions**	**No. of countries**
Develop a research communication and translation platform (guidance notes) to facilitate the use of research findings for policy (decision making), practice and product development	16
Establish a research database to serve as a platform for information sharing amongst all stakeholders to avoid duplication of research	2
Involve policymakers, partners, the private sector and diaspora community in health research	2
Create individual and institutional awards for research	1
Develop tools for collecting data from programmes	1
Establish a national health research management forum to facilitate dialogue on and discussion of research	1
**International community actions**	**Number of countries**
Facilitate science publishing, communication and knowledge translation including sharing of best practices	7
Invite health research staff to regional and international conferences	6
Advocate with the Ministry of Health for the establishment of a national health research forum to facilitate sharing of research findings	6
Support free access to health research journals through initiatives such as HINARI	1
Support government and health research institutions to link research findings to industry	1

Second, a national research database for collating and registering R4H studies conducted in the country needs to be created to facilitate coordination and information sharing amongst stakeholders to avoid duplication of research. This may partly entail developing tools for collecting information pertaining to published and unpublished research from individuals, programmes, institutions and organizations that conduct R4H. Third, a national health research management forum needs to be created to facilitate dialogue on and discussion of research by stakeholders such as policymakers, the media, the community, the industry and researchers. Fourth, policymakers, partners, private sector and the diaspora communities should be involved in the actual R4H production process. Fifth, national awards need to be created for individuals and institutions excelling in R4H. The individual or institutional awards might be granted to nationals or institutions with the most articles in peer-reviewed journals with impact factor rating or to nationals whose research outputs have influenced decision-making or led to the development of new products or ways of improving intervention coverage.

The actions recommended for the international community in stimulating production and use of research findings were to (1) facilitate scientific publishing, communication and knowledge translation, including sharing of best practices, (2) invite national researchers to regional and international R4H conferences to share their research, hone their skills and network, (3) to advocate with the MoHs for the establishment of a national health research forum to facilitate sharing of research findings with all stakeholders, and (4) promote health, preventing disease infections and controlling disease.

### Financing of R4H

Table 7 shows the actions recommended for governments and the international community for reinforcing R4H financing. The recommendations for national governments were to demonstrate more commitment to health research by implementing or advocating for implementation of the research agreements made in Mexico, Abuja, Accra and Bamako to create a R4H budget line in the national budget documents and to increase the R4H budget allocated and disbursed to at least 2 % of the MoH budget. To sustainably honour their commitments to R4H, national governments may need to explore the feasibility of using innovative financing mechanisms such as dedicated tobacco, alcohol, mobile telephone airtime, and road and air travel taxes for research. Given the scarcity of funding for R4H, it is imperative that all research resources and finances be allocated and used efficiently, irrespective of whether the source is public, private, domestic or external. National governments ought to consider the development of mechanisms for monitoring or assessing the allocative and technical efficiency of R4H resources and implementing appropriate ameliorative actions. Some of these mechanisms are discussed exhaustively in the report of the WHO Consultative Expert Working Group on Research and Development: Financing and Coordination [34,35].

**Table 7:** **Actions recommended for national governments and international community for reinforcing R4H financing**

**Actions for countries/national governments**	**Number of countries**
Create a budget line/research fund or raise its level to at least 2% of the national Ministry of Health budget, and ensure actual disbursement of the funds	27
Demonstrate more commitment to health research by implementing and/or advocating for implementation of the research agreements at the Mexico, Abuja, Accra and Bamako forums	6
**Actions for the international community**	**Number of countries**
Provide financial support for strengthening national health research systems to raise the support level and to align the systems with the requirements of the Algiers Declaration, and create a fund for research	23
Implement the recommendation of allocating 5% of development aid project funding to health research	10
Support local NGOs conducting research	1
Promote public-private partnerships to fund health research	1

Four financing-related actions were suggested for the international community. The first was the need to provide, increase and align financial support for strengthening NHRS in line with the Algiers Declaration. This may involve supporting national governments to create a national fund for research and ensuring that all international community research funding is channelled through that fund to implement the priority national health research agenda. The second is to implement the Algiers Declaration and Bamako Call to Action recommendations of allocating 5 % of their development aid project funding to health research. The third is to use the international community’s clout to promote public-private partnerships to fund health research. Fourth, to provide financial support to local NGOs that conduct research within the priority national health research agenda.

### Limitations of the study

- The national health research focal person in each country had the responsibility of completing the questionnaire, working in consultation with government and non-governmental institutions in the country relevant to the aspect of the questionnaire. We do not know how many people were interviewed in those institutions in the process of completing the questionnaire.
- The questionnaire did not assess the numbers of articles published in each country. Questions on research productivity were not included because we were aware that a comprehensive bibliometric study had been funded by the WHO Regional Office for Africa [33].
- The study did not assess the availability of inputs other than information, communication and technology research inputs such as laboratories and reagents or the adequacy of office buildings. It did not adopt a broad definition on the utilisation of research findings for biomedical research and development (product, research and process innovation), and therefore it did not make any enquiries on that.
- We did not try to establish if the funding source had any impact on knowledge translation or capacity building. However, we know from the Jones et al. [36] scoping study of donor approaches to research capacity strengthening in Africa that “*Overall our findings suggest that research capacity support* [by donors] *is focused largely on knowledge generation within universities and research networks, but with little attention to the design of questions that resonate with national policy and development agendas, nor with support for conducting and communicating policy research*”.
- This study did not gather information on the research priority setting approaches used by countries that reported having a national health research priority list. Rudan et al.’s [37] essay on evidence-based priority setting for healthcare and research presents the available tools for priority setting that could be used by policymakers in low-resource settings. In addition, they assessed the applicability and strengths of different tools in the context of maternal and child health in sub-Saharan Africa. Their essay notwithstanding, there will be a future need for a critical analysis of the priority setting approaches applied at least by the 57 % of countries that reported, herein, that they had a national health research priority agenda.

## Conclusion

This study assessed the status of some aspects of NHRS in all the 47 countries of the WHO African Region, identified the factors that enable and constrain NHRS, and generated respondents’ ideas on the way forward. A comparison of the current study’s results with those from previous NHRS surveys in the Region reveals that there has been some improvement in the NHRS landscape – at least in some countries. As the African Region transitions from the Millennium Development Goals to the post-2015 health-related Sustainable Development Goals, it is in dire need of reliable contextual evidence from research to buttress its planning (development of costed roadmaps) and implementation (and monitoring and evaluation) of cost-efficient strategies and interventions. Clearly, the evidence would only be forthcoming if each country’s NHRS has the capacity to effectively and efficiently perform its functions. The 23 (49 %) countries that said that that they did not have an NHRS will need to establish one urgently. For countries that have an NHRS, there is no room for complacency; they need to take the action to reinforce or revamp their NHRS and, if necessary, to re-engineer them to bolster their performance.

## Additional file

Additional file 1:
**Governance structures for health research in the WHO African Region.** (DOCX 21 kb)

## Abbreviations

DALY: Disability-adjusted-life-year
MoH: Ministry of health
MoU: Memorandum of understanding
NGO: Non-governmental organization
NHRS: National health research system
R4H: Research for health
R&D: Research and development
