# Supplementary material for: National health research systems in the WHO African Region: current status and the way forward
Source: Health Res Policy Syst. 2015 Oct 30;13:61. doi: 10.1186/s12961-015-0054-3 (PMC4628337; doi:10.1186/s12961-015-0054-3)
Supplement: Additional file 1: — Governance structures for health research in the WHO African Region. (DOCX 21 kb) [file 12961_2015_54_MOESM1_ESM.docx]

**Additional File 1: Governance structures for health research in the WHO African Region**

| **Country** | **NHP** | **NSHP** | **NHRP** | **HRL** | **NSHRP** | **HRPR** |
| --- | --- | --- | --- | --- | --- | --- |
| Algeria | 1 | 1 | 1 | 1 | 0 | 1 |
| Angola | 1 | 1 | 0 | 0 | 1 | 1 |
| Botswana | 1 | 1 | 1 | 0 | 1 | 1 |
| Burkina Faso | 1 | 1 | 1 | 1 | 1 | 1 |
| Burundi | 1 | 1 | 0 | 0 | 0 | 0 |
| Benin | 1 | 1 | 1 | 1 | 1 | 0 |
| Chad | 1 | 1 | 0 | 0 | 0 | 0 |
| Cameroon | 1 | 1 | 0 | 0 | 1 | 0 |
| Cape Verde | 1 | 1 | 1 | 1 | 0 | 0 |
| Central African Republic | 0 | 1 | 0 | 0 | 1 | 0 |
| Comoros | 1 | 1 | 0 | 0 | 0 | 1 |
| Congo | 1 | 1 | 0 | 0 | 0 | 0 |
| Côte d'Ivoire | 1 | 1 | 1 | 0 | 0 | 0 |
| Democratic Republic of the Congo | 1 | 1 | 0 | 0 | 0 | 0 |
| Eritrea | 1 | 1 | 1 | 0 | 1 | 1 |
| Ethiopia | 1 | 1 | 1 | 0 | 1 | 1 |
| Equatorial Guinea | 1 | 0 | 0 | 0 | 0 | 0 |
| Gabon | 1 | 1 | 0 | 0 | 0 | 0 |
| Ghana | 1 | 1 | 0 | 0 | 1 | 1 |
| Gambia | 1 | 1 | 1 | 0 | 1 | 1 |
| Guinea | 1 | 1 | 1 | 1 | 1 | 0 |
| Guinea-Bissau | 1 | 1 | 0 | 0 | 0 | 0 |
| Kenya | 1 | 1 | 0 | 1 | 0 | 1 |
| Lesotho | 1 | 1 | 1 | 1 | 1 | 0 |
| Liberia | 1 | 1 | 0 | 0 | 0 | 1 |
| Madagascar | 1 | 0 | 1 | 0 | 0 | 1 |
| Malawi | 1 | 1 | 0 | 1 | 0 | 1 |
| Mali | 1 | 1 | 1 | 0 | 1 | 1 |
| Mauritania | 1 | 1 | 0 | 0 | 0 | 0 |
| Mauritius | 1 | 1 | 0 | 1 | 0 | 0 |
| Mozambique | 0 | 1 | 1 | 0 | 0 | 0 |
| Namibia | 1 | 0 | 1 | 1 | 0 | 0 |
| Niger | 1 | 1 | 1 | 1 | 1 | 0 |
| Nigeria | 1 | 1 | 0 | 0 | 1 | 1 |
| Rwanda | 1 | 1 | 1 | 1 | 1 | 1 |
| Sao Tome and Principe | 1 | 1 | 0 | 0 | 0 | 0 |
| Senegal | 1 | 1 | 1 | 1 | 1 | 1 |
| Seychelles | 1 | 1 | 0 | 0 | 0 | 0 |
| Sierra Leone | 1 | 1 | 1 | 0 | 0 | 1 |
| South Africa | 1 | 1 | 1 | 1 | 1 | 1 |
| South Sudan | 1 | 1 | 0 | 0 | 0 | 0 |
| Swaziland | 1 | 1 | 1 | 0 | 1 | 1 |
| Tanzania | 1 | 1 | 1 | 1 | 1 | 1 |
| Togo | 1 | 1 | 0 | 1 | 0 | 0 |
| Uganda | 1 | 1 | 1 | 1 | 1 | 1 |
| Zambia | 1 | 1 | 0 | 1 | 1 | 1 |
| Zimbabwe | 1 | 1 | 0 | 1 | 0 | 1 |

Key:

1 = Yes; 0 = No

NHP = national health policy, NSHP = national strategic health plan, NHRP = national health research policy, NSHRP = national strategic plan for health research, HRPR = health research programme, HRL = health research law
